# Complete mitochondrial genome sequence of the Asiatic Toad *Bufo Gargarizans* (Amphibia, Anura, Bufonidae)

**DOI:** 10.1080/23802359.2017.1407700

**Published:** 2017-11-25

**Authors:** Lichun Jiang, Yabin Liu, Li Zhao, Qiping Ruan

**Affiliations:** a Key Laboratory for Molecular Biology and Biopharmaceutics, School of Life Science and Technology, Mianyang Normal University, Mianyang, Sichuan, PR China;; b Department of Life Science and Engineering, Jining University of Shandong, Qufu, Shandong, PR China

**Keywords:** Bufo gargarizans, Bufonidae, complete mitochondrial genome, protein-coding genes

## Abstract

The Asiatic toad *Bufo gargarizans* belongs to Bufonidae. This species is known from the Russian Far East, central, northern and north-eastern China, the Democratic People's Republic of Korea and Japan. In this study, the complete mitochondrial genome of *B. gargarizans* was sequenced. The mitogenome was 17,407 bp in length, consisting of 13 protein-coding genes, 22 transfer RNA (tRNA) genes, two ribosomal RNA (rRNA) genes, and a non-coding control region. As in other vertebrates, most mitochondrial genes are encoded on the heavy strand, except for ND6 and eight tRNA genes which are encoded on the light strand. The overall base composition of the *B. gargarizans* is 28.9% A, 28.2% T, 27.5% C, and 15.3% G. Phylogenetic analysis showed *B. gargarizans* was closely related to *B. bankorensis* and *B. tibetanus.* The complete mitogenome of *B. gargarizans* can provide an important data for the studies on phylogenetic relationship and population genetics to further explore the taxonomic status of this species.

The Asiatic toad *Bufo gargarizans* belongs to Bufonidae, which is one of the most species-rich amphibian families (Fei et al. 2009). This species is a species of toad endemic to East Asia and portions of the Russian Far East, but relatively rare on the Korean Peninsula (Kuzmin et al. 2004; Kuzmin 2015). *Bufo gargarizans* is an important model species in Bufonidae and is common in China (Cao et al. 2006). The Asiatic toad plays an important role in traditional Oriental medicine. A novel antimicrobial peptide was isolated from the stomach tissue of *Bufo bufo gargarizans* and showed more potent antimicrobial activities (Park et al. 1996). In this study, we sequenced the complete mitochondrial genome of *B. gargarizans* (17,407 bp; GenBank accession no. KU321581), with the individual sampled from the Mojia town, Mianyang city, Sichuan province, China in June 2014 (104°32′E and 31°25′N; 492 m elevation), and the specimen is stored in the Key Laboratory for Molecular Biology and Biopharmaceutics from Mianyang Normal University. We employed polymerase chain reaction (PCR) and Long-and-Accurate PCR methods to amplify the whole mitogenomic region of *B. gargarizans* with the PCR primers designed by Kurabayashi and Sumida (2009) and ourselves. The reaction protocol, amplification system and sequencing saw Kurabayashi and Sumida’s (2009) and Jiang’s methods (Jiang 2013).

The complete mitochondrial genome of *B. gargarizans* is similar to those of other vertebrates' mitochondrial genomes including 13 typical protein-coding genes, 22 transfer RNA (tRNA) genes, two ribosomal RNA (rRNA) genes, and one control region (CR). The overall base composition is 28.9% A, 28.2% T, 27.5% C, and 15.3% G, with an AT content of 57.1%, which agreed with the trend of most mitochondrial genomes. As in other vertebrates, most mitochondrial genes are encoded on the heavy strand, except for ND6 and eight tRNA genes, which are encoded on the light strand. All 13 mitochondrial protein-coding genes share the start codon ATG, except for ND1, ND2, and COXI (ATT and ATA start codon, respectively). It is also important to note that the majority of protein-coding genes (five of 13 genes) are inferred to terminate with an incomplete stop codon T–– (ND1, ND2, COXII, COXIII, and ND3); five protein-coding genes share the typical termination codon TAA (COXI, ATP8, ATP6, ND4L, and ND4); ND5, ND6, and Cyt b use AGA as a stop codon (Table 1). For 13 protein-coding genes, the A + T content ranges from 50.91% to 57.58%, which is higher than that of G + C. In the complete mitochondrial genome, there are totally 47 overlapped nucleotides between neighbouring genes in 10 locations and the length of overlapped sequence is 1–17 bp, while there are totally 66 bp intergenic nucleotides in 9 locations and the length of intergenic spacer is 1–37 bp. Twenty pairs of genes are directly adjacent without intergenic or overlapping nucleotides (Table 1). The two ribosomal RNA genes, 12S rRNA (935 bp) and 16S rRNA (1599 bp), are located between tRNA^Phe^ and tRNA^Leu^ (UUR) and separated by tRNA^Val^ gene, and the lengths of 22 tRNA genes range from 64 to 73 bp (Table 1). The putative origin of light-strand replication (O_L_) (29 bp in length), as in most vertebrates, is located in a cluster of the tRNA^Trp^–tRNA^Ala^–tRNA^Asn^–tRNA^Cys^–tRNA^Tyr^ region (WANCY region). The other non-coding region, the putative control region (2000 bp in length), was bound by tRNA^Leu^ and Cyt *b* (Table 1).

**Table 1.** Characteristics of the mitochondrial genome of *Bufo gargarizans*.

	Position			Codon			
Gene	From	To	Sizes nudeotide (bp)	Start	Stop^a^	Intergenic nudeotide^b^	Strand^c^
tRNA-Leu	1	72	72				H
tRNA-Thr	73	144	72			−1	H
tRNA-Pro	144	212	69			−1	L
tRNA-Phe	212	279	68				H
12S ribosomal RNA	280	1214	935			−5	H
tRNA-Val	1210	1278	69				H
16S ribosomal RNA	1279	2877	1599			1	H
tRNA-Leu	2879	2951	73			15	H
ND1	2967	3912	946	ATT	T––		H
tRNA-Ile	3913	3983	71			−1	H
tRNA-Gln	3983	4053	71			−1	L
tRNA-Met	4053	4121	69				H
ND2	4122	5154	1033	ATA	T––		H
tRNA-Trp	5155	5224	70				H
tRNA-Ala	5225	5293	69				L
tRNA-Asn	5294	5366	73				L
L-strand origin of replication	5367	5395	29			−3	L
tRNA-Cys	5393	5456	64				L
tRNA-Tyr	5457	5526	70			4	L
COXI	5531	7072	1542	ATA	TAA	2	H
tRNA-Ser	7075	7145	71			1	L
tRNA-Asp	7147	7215	69			1	H
COXII	7217	7904	688	ATG	T––		H
tRNA-Lys	7905	7976	72			1	H
ATP8	7978	8142	165	ATG	TAA	−10	H
ATP6	8133	8816	684	ATG	TAA	−1	H
COXIII	8816	9599	784	ATG	T––		H
tRNA-Gly	9600	9668	69				H
ND3	9669	10,008	340	ATG	T––		H
tRNA-Arg	10,009	10,077	69				H
ND4L	10,078	10,377	300	ATG	TAA	−7	H
ND4	10,371	11,735	1365	ATG	TAA		H
tRNA-His	11,736	11,804	69				H
tRNA-Ser	11,805	11,871	67			37	H
ND5	11,909	13,711	1803	ATG	AGA	−17	H
ND6	13,695	14,189	495	ATG	AGA		L
tRNA-Glu	14,190	14,257	68			4	L
Cyt b	14,262	15,407	1146	ATG	AGA		H
Control region	15,408	17,407	2000				H

^a^ T–– represent incomplete stop codons.

^b^ Numbers correspond to the nucleotides separating adjacent genes and negative numbers indicate overlapping nucleotides.

^c^ H and L indicate genes transcribed on the heavy and light strands, respectively.

The three gene (12S rRNA, 16S rRNA, and Cyt *b*) sequences were aligned by MEGA 6.0 (Tamura et al. 2013) with the default settings. The concatenated sequence data of 12S rRNA, 16S rRNA, and Cyt *b* genes of *B. gargarizans* and other 13 *Bufo* species were used for the phylogenetic analysis, with setting the *Atelopus bomolochos* and *Atelopus varius* as outgroups. Phylogenetic analysis based on three gene sequences using the neighbour-joining (NJ) method showed that *B. gargarizans* was closely related to *B. bankorensis and B. tibetanus* (Figure 1). Though the support values of our phylogenetic trees are high, many complete mitogenome sequences within genus *Bufo* are still deficient. Therefore, more mitogenomic data are required to better understand the phylogenetic relationship within *Bufo*. Thus, the *B. gargarizans* mitogenome sequence can expand phylogenetic knowledge of the genus *Bufo*.

**Figure 1.**
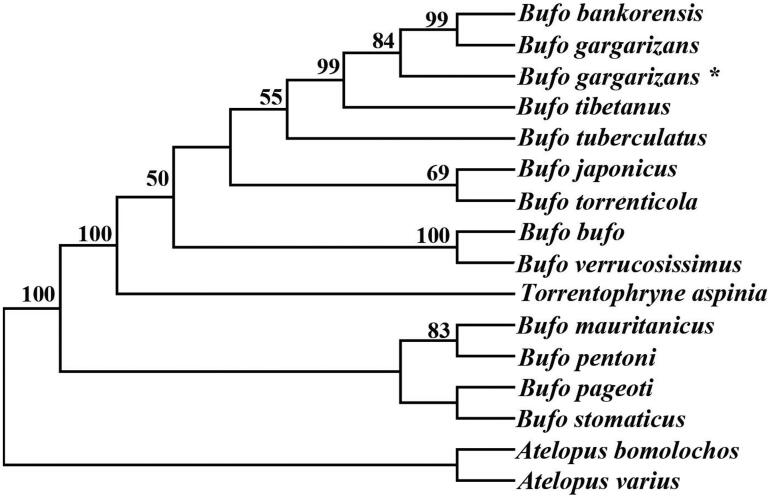
Phylogenetic trees of *Bufo* species reconstructed from concatenated DNA sequences of three genes on heavy-strand. Tree topologies produced by neighbour-joining (NJ). *Atelopus bomolochos* and *Atelopus varius* were used as outgroups. Bootstrap support (50%) based on 1000 replicates are shown at nodes. The asterisk indicates the sequence generated in this study.
